# Healthcare experiences of transgender and gender-diverse people in the UK: a scoping review

**DOI:** 10.1136/bmjopen-2025-106519

**Published:** 2026-05-04

**Authors:** Lauren Davidson, Aiden Moore, Amy Grove, Julia Gauly

**Affiliations:** 1 Warwick Medical School, University of Warwick, Coventry, UK; 2 Liverpool University Hospitals NHS Foundation Trust, Liverpool, UK; 3 Centre for Evidence and Implementation Science, University of Birmingham, Birmingham, UK

**Keywords:** Transgender Persons, Health Services, Health Services for Transgender Persons, Sexual and Gender Minorities

## Abstract

**Abstract:**

**Objectives:**

To map the existing literature on transgender and gender-diverse (TGD) people’s experiences of healthcare in the UK, summarising key findings and characteristics of current research.

**Design:**

This scoping review was guided by the Arksey and O’Malley framework and reported in line with Preferred Reporting Items for Systematic Reviews and Meta-Analyses extension for Scoping Reviews (PRISMA-ScR) guidelines.

**Data sources:**

MEDLINE, Embase, PsycINFO and Web of Science were searched on 29 October 2024. Reference lists of included studies were searched to identify further relevant literature.

**Eligibility criteria for selecting studies:**

Primary research of any design reporting the healthcare experiences of TGD people in the UK from 2019 onwards was included.

**Data extraction and synthesis:**

Relevant data were extracted using a data charting form guided by the research questions and the Arksey and O’Malley framework. Results were narratively synthesised to provide a comprehensive overview of the literature.

**Results:**

52 studies were included, comprising qualitative (n=38), mixed-methods (n=12) and quantitative (n=2) designs. TGD people reported varying experiences across general and transition-related healthcare settings. Positive experiences were often attributed to individual clinicians. Negative experiences were common, including transphobic discrimination, insufficient clinician knowledge and barriers to effective transition-related care. Strategies for navigating healthcare, such as information sharing within the TGD community and educating clinicians about trans health needs, reflected adaptive responses to systemic barriers.

**Conclusion:**

The findings highlight a need for greater recognition of diverse gender identities, more inclusive approaches in healthcare education and practice and further targeted research. This is especially urgent in light of the recent UK Supreme Court judgement regarding definitions of ‘sex’ under the Equality Act 2010, the emerging implications of which risk compounding existing barriers by reinforcing exclusionary practices within healthcare settings and further limiting recognition and protections for TGD people in the UK.

**STRENGTHS AND LIMITATIONS OF THIS STUDY**

- The review was guided by the established five-stage Arksey and O’Malley framework and reported in accordance with the Preferred Reporting Items for Systematic Reviews and Meta-Analyses extension for Scoping Reviews (PRISMA-ScR), ensuring methodological rigour and transparency.
- Additional database searches, hand-searching and a formal grey literature search could have enhanced the comprehensiveness of the review.
- Evolving terminology around transgender and gender-diverse topics posed challenges in identifying all relevant literature.
- Variability in study quality and the absence of critical appraisal may limit the reliability and contextual interpretation of findings.

## Introduction

 Transgender and gender-diverse (TGD) is an inclusive umbrella term encompassing people whose gender identity does not align with sex assigned at birth, as well as those with gender identities or expressions that exist outside or beyond binary classifications.^1 2^ The healthcare experiences of this population are complex, influenced by the enduring pathologisation of their identities, pervasive social stigma and intersecting systems of oppression, such as racism, ableism and classism.^3 4^ In the UK, evidence suggests that TGD people face specific barriers to accessing healthcare, including avoiding treatment for fear of discrimination or intolerance, as well as poorer health outcomes.^5 6^

Patient experience, alongside patient safety and clinical effectiveness, is a core component of healthcare quality improvement under the Health and Social Care Act 2012^7^ with research indicating consistent positive associations between these dimensions across diverse outcomes and settings.^8^ Understanding the healthcare experiences of marginalised communities is therefore essential to reducing health inequalities and improving the quality of their care.^9^

Several recent literature reviews have explored TGD healthcare experiences in various areas, including primary care,^10^ emergency departments,^11^ gender-affirming care,^12^ cancer care,^13^ sexual and reproductive healthcare^14^ and among children and young people^15 16^; all included studies from multiple countries. While international data can offer valuable insights, findings may not be generalisable across contexts due to variations in the legal rights, societal acceptance and provision of healthcare for TGD people worldwide. Changes made to the provision of puberty blockers in the UK following the Cass Review in April 2024^17 18^ exemplify how healthcare for TGD populations is shaped by national policy and emerging evidence, even when that evidence may be limited or disputed.^19 20^

We aimed to understand what is known about the healthcare experiences of TGD people in the UK by conducting a scoping review to systematically identify and map existing evidence.^21^ Scoping reviews are well suited to broad and evolving topics such as TGD healthcare experiences, which reflect shifting social attitudes and span multiple settings. This approach was preferred over a systematic review, as it facilitates the identification of knowledge gaps, providing insights to inform future research and targeted evidence syntheses.^21^

Our review addressed the following research questions:

What is the extent, range and nature of existing literature reporting how TGD people experience the delivery of healthcare in the UK?

What is known from the existing literature about the healthcare experiences of TGD people in the UK?

These questions were developed using the PICo (Population, phenomena of Interest, Context) framework^22^ and guided by the Arksey and O’Malley framework for scoping reviews.^23^ PICo was chosen due to the focus on the lived experiences (phenomena of interest) of the population (TGD people) within a specific context (UK healthcare).

## Methods

This review was guided by the five-stage Arksey and O’Malley framework and is reported in accordance with the Preferred Reporting Items for Systematic reviews and Meta-Analyses extension for Scoping Reviews (PRISMA-ScR)^24^ (see online supplemental file). The review protocol was registered with the Open Science Framework and is available at: https://doi.org/10.17605/OSF.IO/JCZK.

### Information sources and search

Database selection was carefully considered in consultation with a specialist librarian to provide comprehensive coverage of clinical (Embase, MEDLINE), psychological (PsycINFO) and multidisciplinary (Web of Science) research. Initial search strategies were developed by the first and last author, informed by the research questions and previous reviews conducted in this field^10 12 15^ and subsequently reviewed by a specialist librarian to ensure that key concepts, terms and search syntax were appropriately applied.

All database searches were conducted by the first author on 29 October 2024. Search terms were adapted for each database, combining relevant free-text and Medical Subject Heading terms using the Boolean operators (AND), (OR) and (NOT). Truncation techniques were used to capture variations in spelling and enhance retrieval. Searches were limited to records published from 1 January 2019 to the most recent records available at the time of searching. This decision was informed by the publication of the eleventh revision of the International Classification of Diseases (ICD-11) in May 2019, which reclassified terms related to gender diversity.^25^ In ICD-10, the terms ‘transsexualism’ and ‘gender identity disorders’ are listed within ‘Disorders of adult personality and behaviour’.^26^ These terms do not appear in ICD-11, which introduces ‘gender incongruence’ within a new category, ‘Conditions related to sexual health’ and extends recognition to the experiences of children and adolescents.^25^ The updated classification marks a significant shift in the medical understanding of trans and gender-diverse identities. Geographical search terms were informed by the National Institute for Health and Care Excellence (NICE) UK filters to optimise identification of studies conducted in the UK.^27^ Additional publication type limits were applied where available to align with the inclusion of primary research studies only. Full search strategies for all databases are provided in online supplemental file 1. Due to time and resource constraints, a comprehensive grey literature search was not feasible. To partially address this, reference lists of all included studies were screened to identify additional relevant sources beyond the indexed literature.

### Eligibility criteria

Primary research studies reporting the first-hand healthcare experiences of TGD people in the UK were eligible for inclusion. Studies published prior to 2019 were excluded to generate findings most relevant to current practice. Specific inclusion and exclusion criteria are detailed in table 1.

**Table 1:** Eligibility criteria

Inclusion criteria	Primary research of any design including qualitative, quantitative and mixed-methods
	Studies reporting the experiences¹ of TGD² people accessing healthcare in the UK, as reported by TGD people themselves ¹Experiences include: service user perceptions of interactions with any healthcare services, perceived barriers and/or facilitators encountered when accessing any healthcare services, the perceived quality and/or efficiency of any healthcare services accessed ²Refers to individuals holding any identity under the TGD umbrella including, but not limited to, transgender men and women, non-binary people and individuals identifying as genderqueer, genderfluid, agender or gender non-conforming
Exclusion criteria	No mention of TGD people as a population or participant focus
	Studies involving TGD people that do not report experiences of healthcare in the UK
	Data relating to treatment outcomes
	Opinion pieces, feature articles, editorials, reviews, book chapters, conference abstracts, protocols and non-primary data
	Publication date prior to 2019
	Duplicate studies
	Studies published not in English
	Studies with no full text available

TGD, transgender and gender-diverse.

### Selection of sources of evidence

Articles identified through database searches were imported into the web-based systematic review software, Rayyan,^28^ where duplicates were detected, screened and removed by one reviewer using Rayyan’s standard (non-artificial intelligence) duplicate detection. Titles and abstracts of the remaining records were independently screened by two reviewers against the eligibility criteria, with discrepancies resolved through discussion or screening by a third reviewer. Full texts of potentially relevant articles were then retrieved and independently screened by two reviewers, with reasons for exclusion recorded. Discrepancies were resolved through discussion or consultation with a third reviewer. Reviewers were blinded to each other’s decisions during both independent screening stages; blinding was removed to identify and resolve discrepancies.

### Data charting

A data charting form was developed based on the research questions, discussion within the review team and the Arksey and O’Malley framework.^23^ During the early stages of data charting, additional relevant data items were identified, specifically community involvement in study design, prompting a revision of the charting form. Data extraction was then completed by the primary reviewer using the finalised form. Table 2 provides an overview of the extracted data items.

**Table 2:** Extracted data items

Publication information	Author(s)
	Title
	Year/journal of publication
Study information	Design, ie, qualitative, quantitative, mixed-methods
	Methods, eg, interview, focus group, survey
	Study objectives
	Community involvement in study design
	Funding
Sample information	Sample size
	Gender identity of participants
	Age of participants
	Other demographic information, eg, ethnicity, level of education (if provided)
Setting	Healthcare setting(s)
	Location(s) within the UK
Key findings	Outcomes of interest, eg, service user outcomes (experiences, barriers, facilitators), perceived quality and/or efficiency of healthcare services accessed

A representative subset of eight (15%) of the included studies was selected to reflect a range of study designs, methods, populations and sample sizes. This subset was independently charted by a second reviewer to assess consistency and accuracy. As no disagreements arose during this independent extraction, all remaining studies were subsequently checked and revised by a second reviewer rather than undergoing full independent extraction. This ensured accuracy while streamlining the process. A final review of the dataset was conducted by the primary reviewer prior to synthesis.

### Synthesis of results

Extracted data were analysed and summarised to provide a comprehensive overview of the literature, with results presented in tables and narrative summaries. Basic numerical analyses were conducted to summarise the distribution and characteristics of included studies, including research methods, population groups, healthcare settings and geographical location. Thematic categories derived from charted data were developed iteratively by the primary reviewer and refined through discussion with the wider team to ensure consistency and transparency. Key findings were initially grouped into 25 granular categories reflecting specific aspects of healthcare experiences (eg, gender service waiting times, transphobic discrimination, positive experiences, self-advocacy). These categories were then reviewed and consolidated into three overarching thematic categories: positive experiences, negative experiences and experiences of navigating healthcare. The narrative presentation reflects a structured descriptive summary of the included studies, consistent with scoping review methodology.^23^ As critical appraisal of individual sources of evidence was beyond the scope of this review, no quality assessment was performed.

## Results

### Selection of sources of evidence

**Figure 1:**
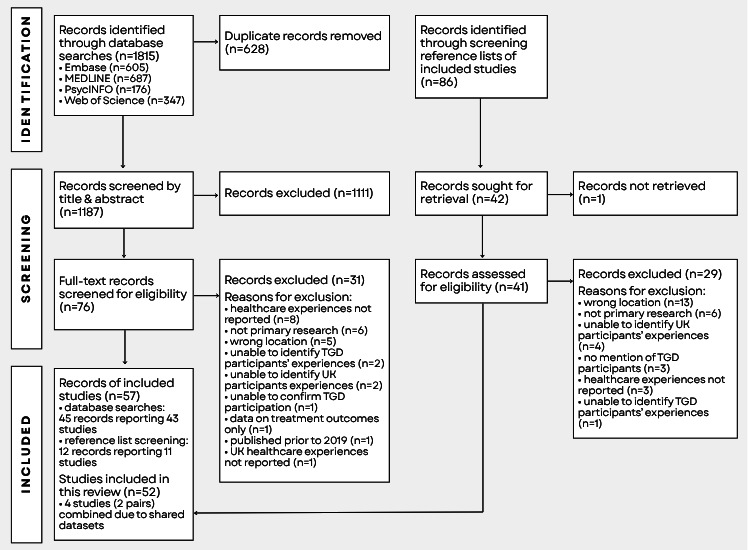
PRISMA flow diagram. PRISMA, Preferred Reporting Items for Systematic reviews and Meta-Analyses; TGD, transgender and gender-diverse.

Of 1187 records identified through database searches, 76 full texts were retrieved following title and abstract screening. Of these, 43 unique studies were eligible for inclusion. Reference list screening of included studies identified a further 11 unique studies eligible for inclusion. Data charting identified two results reporting overlapping and distinct data from the same participant sample^29 30^ and two results drawing from the same dataset,^31 32^ which have been combined for the purposes of this review. A further two results which drew from the same participant sample have been retained as separate studies due to their distinct objectives and analytical focus.^33 34^ Accounting for these combinations, a total of 52 studies were included in the synthesis. The selection process and reasons for exclusion following full-text screening are presented in figure 1.

### Characteristics of sources of evidence

Included studies employed qualitative (n=38), mixed-methods (n=12) and quantitative (n=2) designs. The most common methods of primary data collection were interviews (n=34), surveys (n=12) and workshops or focus groups (n=10). Almost all studies (n=48) involved UK-based participants only, although 25 of these did not report the specific location of participants within the UK.

Transition-related care settings, defined for the purpose of this review as services providing care specifically related to gender transition, were most prevalent (n=33). These included specialist gender services accessed privately (n=10) or via NHS (National Health Service) pathways (n=32), as well as transition-related interactions within general practice (n=16) or mental health services (n=4). Experiences unrelated to transition were reported across various healthcare settings, including mental health services (n=14), general practice (n=14), sexual health services (n=6), fertility services (n=6), hospitals (n=5), screening services (n=5), perinatal services (n=4), gynaecology services (n=2), pharmacy services (n=1) and prison healthcare (n=1). 24 studies reported experiences where the healthcare setting was unclear.

The majority of studies (n=39) focused specifically on TGD populations, of which 22 reported some form of TGD community involvement in the design and/or conduct of the study. This included TGD research team members (n=9) and/or consultation with TGD individuals or engagement groups (n=12) or TGD-led charities (n=4). A further four studies reported involvement of LGBT+community members without specifying whether this included TGD people. 13 studies did not report any direct involvement of TGD or LGBT+individuals. The characteristics of included studies are presented in online supplemental file 2.

### Synthesis of results

Participants reported a range of experiences across healthcare settings, broadly grouped into three thematic categories: positive experiences, negative experiences and experiences of navigating healthcare.

#### Positive experiences (n=25)

Across healthcare settings, positive experiences were predominantly attributed to individual clinicians. Participants valued supportive and reassuring practitioners,^31^,^44^ as well as those who proactively sought information to better support their TGD patients.^3134 40 45^,^47^

At gender clinical appointments, participants appreciated having time to discuss their concerns^38 40 48^ and the thorough assessments provided.^49^ Adaptations to accommodate autistic participants’ communication and sensory needs were also valued.^50 51^ Participants in two studies reported initial feelings of ‘hope’^42^ and ‘euphoria’^52^ on being referred to specialist services. Squires *et al*^52^ also highlighted three participants who found services to be responsive and reassuring during the referral process. In five studies, participants described the benefits of accessing private transition-related services, noting prompt and reliable access.^34 39 40 47 53^

In general healthcare settings, the proactive use of inclusive language was identified as a facilitator of respectful and affirming care,^34 40 41 47 54^ reducing the burden on TGD patients to advocate for their needs: ‘my care providers told everyone ahead of time, [saying), ‘this is the language that Adam uses’’.^41^ Two participants appreciated being allocated private rooms when accessing gynaecology^42^ and perinatal^55^ hospital services. One study reported positive sentiment associated with the human-free nature of chatbot-assisted self-referral to mental health services.^56^

#### Negative experiences (n=49)

Negative experiences were common across healthcare settings. Participants seeking transition-related care through the NHS reported difficulties securing referrals from their GPs (general practitioners), citing limited awareness of referral pathways^31 34 40 42 45 47 57^ or an unwillingness to refer.^40 45 47 51 57^ For those successfully referred, long waiting times were a prominent concern. Participants described how delays negatively impacted their mental health,^3439 40 42 45 47 48 52 58^,^60^ as well as the lack of interim support while waiting for appointments.^42 47 52 59 61^ Others reported feeling ‘forgotten about’^39 53^ or that ‘nobody cares’.^44^ Experiences of waiting times were particularly distressing for individuals approaching or experiencing puberty^45 58^ and for those trying to access transition-related interventions later in life.^32 40 47 62^ Several studies reported that participants had either accessed^34 39 40 42 47 50 59 63^ or were considering^31 39 52 63^ private gender services in response to prolonged NHS waiting times.

While perceptions of waiting were largely negative, two participants described how the long waiting period allowed time for ‘thinking and working things out’^40^ or becoming more comfortable with themselves: ‘I’m going to just go on with me life and be who I am’.^52^

Communication issues concerning gender services were identified across 15 studies, in which participants reported poorly communicated cancellations,^31 42 60 64^ difficulties in contacting services^39 40 42 47 50 52 65^ and extended periods of non-communication while waiting.^32 34 39 42 47 48 52 58 59 65^ Negative experiences also arose from poor communication between service providers, such as necessary test results being unavailable at appointments^40^ or waiting months for letters about ongoing care to be sent to their GP.^31^

For participants who had accessed specialist services, either privately or via NHS pathways, further challenges included GPs reluctant to enter into shared care agreements^40 45 47^ or inadequate management of hormonal therapy.^31 32 40 47^ Individuals sourcing hormonal medication through unregulated providers reported being denied monitoring tests and/or bridging prescriptions by their GP.^40 42 47 52^

Negative experiences were commonly associated with a lack of understanding of TGD identities among healthcare providers.^29^,^3134^ This was particularly evident among participants with intersecting marginalised identities, such as people with disabilities or chronic illness,^46 50 51^ and black people and people of colour.^40 43 55 69^ One participant noted that they ‘encountered quite a few professionals in the medical field who don’t understand what it means [to be both TGD and autistic]’,^46^ while another highlighted how ‘existing at the intersections of both like race and gender […] [means] that your care is going to be quite insufficient for what you need’.^55^ In a study focusing on transgender sex workers, several participants reported not feeling comfortable contacting a GP or being unable to access healthcare they believed to be medically necessary.^73^

Negative experiences were also associated with questioning that felt invasive or lacked appropriate context in both transition-related^29 30 40 45 47 59^ and general healthcare settings.^4044 47 50 74^,^76^ Examples included questions about gender identity which felt irrelevant to the consultation,^44 47 50 74 75^ and younger participants distressed by discussing sensitive topics with ‘strangers’ during gender clinical appointments.^29^ Another participant described repeated questioning about trauma in relation to their physical health without clear contextualisation: ‘trauma tends to be something they ask you with a lot of the conditions I have […] I don’t know whether it’s just coincidence or what’.^76^

Transphobic discrimination in medical settings was reported in 19 studies.^3133^,^35 40^ This included unfair treatment in general healthcare (n=13), such as having symptoms incorrectly attributed to hormonal treatments—‘Whenever you go to the GP about anything, the GP is always like, ‘it’s your hormones’ […] for everything’^42^—or being told they were ‘too complicated to deal with’ by sexual health services.^44^ Misgendering and deadnaming were reported in both transition-related (n=5) and non-transition-related settings (n=9). Other examples of enacted prejudice included being ‘recommended religion as conversion treatment’^35^ or told ‘that being trans is just due to trauma’.^45^

Additional systemic barriers to inclusive care were reported across healthcare settings. Negative experiences were associated with the gendered organisation of care in specific contexts, including hospital wards,^40 42 47^ perinatal care,^41 55 74^ screening programmes,^35 40 42 47^ gynaecological care^78^ and fertility^66^ and sexual health services.^33 44^ Participants also described the negative impact of inflexible data collection and record-keeping systems, reporting difficulties updating names and gender markers in medical records,^40 42 45 47 53^ instances of misgendering or deadnaming in documentation and correspondence^31 40 47 57 66 68^ and challenges arising from incongruence between their gender identity and their medical records.^33 40 42 44 79^ These structural issues were of particular concern for participants who did not identify within the gender binary.^3441^,^43 45 47 49 50 55 59 68^

In transition-related settings, participants across the gender spectrum described an expectation to conform to binary norms, often feeling they had to ‘convince’ clinicians of the authenticity of their trans identities to be considered eligible for referrals or interventions.^29^,^3140 42 45 47^ Non-binary individuals reported concealing their identities during interactions with gender clinicians due to concerns that disclosure might compromise their care.^34 42 45 49 50 59^ One participant described being ineligible for laser treatment ‘because you have to tick the whole ‘male to female’ box’.^80^ Others reported concealing mental health difficulties,^42 47 49 59^ trying to ‘appear as neurotypical as possible’,^51^ or forgoing further investigation of intersex traits,^40^ fearing these factors might delay or prevent access. Such experiences contributed to the perception that TGD people had to ‘jump through hoops’ when accessing transition-related care.^30 39 59 69^

Participants also identified geographical and financial barriers to transition-related care, including regional variation in service provision,^31 40 75^ a perceived lack of understanding about TGD identities in rural areas,^40 47^ the unaffordability of private services,^31 40 42 47 53 59^ lengthy travel distances^31 40 42 45^ and the associated travel expenses.^31 40^

Studies reporting the impact of COVID-19 highlighted negative experiences associated with changes in the provision of mental health services,^81^ prison services^60^ and perinatal care.^82^ The pandemic was also reported to have compounded existing waiting times and delays for participants accessing transition-related care.^42 60 62 64 65 72 81^

#### Experiences of navigating healthcare (n=21)

Participants described a range of strategies for navigating unsupportive or inadequate healthcare systems. Five studies identified information sharing within the TGD community as a facilitator of improved care. This included accessing online resources such as discussion forums^47^ and trans GP lists,^53^ as well as advice from other TGD individuals.^40 41 47 53 71^ Further strategies involved participants taking steps to manage their own care by following up on referrals,^31 32 34 40^ correcting administrative errors,^40 42^ or modifying clinical environments to feel more inclusive: ‘I remember just crossing out [gendered] signs in the hospital and putting parent’.^74^

Several additional studies highlighted the importance of self-advocacy. Many participants reported having to educate healthcare professionals about trans health needs^31 33 34 38 40 44 45 47 59 78^ or conducting personal research to compensate for a lack of guidance.^3142 45 47 48 51^,^53 71^ Some described needing to be ‘insistent’,^38 45^ or ‘push’^47^ against the system to have their needs met. These experiences were sometimes framed in combative terms, with participants describing the need to ‘fight’,^31 59 71^ ‘battle’^40 41^ and ‘bulldoze’^47^ their way through, ‘armed’ with information^51^ to support their requests.

Seven studies documented the practical and emotional toll of self-advocacy. One participant identified ‘having to be an expert in [their] own health’ as a barrier to cervical screening,^35^ while another described declining analgesia during childbirth due to concerns they would be unable to advocate for themselves if not ‘fully present’.^55^ Others characterised their experience of self-advocacy as ‘difficult’,^46^ ‘challenging’,^78^ ‘exhausting’^40 47^ or associated with ‘great emotional cost’.^41^

## Discussion

### Summary of evidence

Our findings reveal multiple barriers and systemic shortcomings that contribute to experiences of discrimination and inadequate care for TGD people accessing healthcare in the UK. The strong association of positive experiences with individual clinicians demonstrates the impact of a compassionate, person-centred approach while pointing to inconsistencies in service delivery and care quality. Strategies for navigating healthcare reflect the resilience of TGD individuals and communities in the face of systems that frequently fail to provide appropriate support. Participant experiences spanned a range of healthcare services and settings, with transition care related to gender transition being most commonly explored. The predominance of qualitative studies reflects a focus on capturing nuanced lived experiences; however, the relatively small number of quantitative approaches indicates a lack of generalisable data.

### Limitations

Grey literature was included through reference list screening. However, as no formal grey literature search was conducted, unpublished or non-indexed evidence may have been missed. Specifically, insights from non-academic and advocacy sources may be under-represented, which may limit the comprehensiveness of the review. While the selected databases covered multiple disciplines, searching additional databases and hand-searching key journals could have enhanced coverage. Evolving terminology around TGD topics also poses challenges in identifying all relevant literature. The review team included a queer genderqueer person, a gay cisgender man and two heterosexual cisgender women. We reflected on our individual and collective positionalities throughout, with consideration of how this could influence the interpretation and synthesis of findings. We did not formally assess publication bias or study quality, which may limit the representativeness of our findings.

## Conclusions

To our knowledge, this is the first scoping review on the healthcare experiences of TGD people in the UK. The national focus ensures direct relevance, reflecting experiences shaped by UK-specific healthcare policies, systems and socio-cultural factors. Consistent with reviews by Holland *et al*,^10^ Goulding *et al*^15^ and Chong *et al*,^16^ our findings indicate that TGD people face widespread discrimination when accessing healthcare services. Inadequate knowledge among healthcare providers is consistently reported across the recent literature^10^,^16^ and, as reflected in our review, often results in patients assuming the role of educator during healthcare interactions.^10^,^1214 16^ Taken together, these findings highlight persistent gaps between the care TGD patients report experiencing and the care required to meet their needs, underscoring the importance of improved and sustained professional education addressing the specific health needs of TGD populations.^10^,^15^

Further research exploring barriers that limit healthcare professionals’ ability to effectively support TGD patients could contribute to the existing literature^83 84^ and identify actionable strategies for service improvement. Greater attention should also be given to under-explored contexts, such as pharmacy services, to develop a comprehensive understanding of the full spectrum of TGD healthcare needs. Additionally, existing qualitative insights could be supported by further quantitative research to provide statistical validation of commonly reported experiences.

The need for further research and inclusive healthcare practices is particularly pressing in light of the UK Supreme Court judgement in For Women Scotland v The Scottish Ministers (April 2025).^85^ Although its full impact remains unclear at the time of writing, the judgement may have wide-ranging social and legal implications for the rights and recognition of TGD people in the UK.^86^ This stresses the immediate importance of generating robust, context-specific evidence to inform policy decisions that support gender-diverse populations, alongside ensuring that TGD people are meaningfully involved in shaping research and policies that directly affect them.^87^

## Supplementary material

10.1136/bmjopen-2025-106519 online supplemental file 1

10.1136/bmjopen-2025-106519 online supplemental file 2

10.1136/bmjopen-2025-106519 online supplemental file 3

## Data Availability

All data relevant to the study are included in the article or uploaded as supplementary information.
