# Supplementary material for: Healthcare experiences of transgender and gender-diverse people in the UK: a scoping review
Source: BMJ Open. 2026 May 4;16(5):e106519. doi: 10.1136/bmjopen-2025-106519 (PMC13141246; doi:10.1136/bmjopen-2025-106519)
Supplement: online supplemental file 2 [file bmjopen-16-5-s002.docx]

| **Database** | Embase |
| --- | --- |
| **Coverage** | 1974 to 2024 October 25 |
| **Platform** | Ovid |
|  | |
| **PICo** | **EMBASE Search Terms** |
| **Population** | exp male to female transgender/ or exp female to male transgender/ or exp "transgender and gender nonbinary"/ or exp transgender/ or exp gender diversity/ or exp gender identity/ or exp gender nonbinary/ or exp transsexualism/ or exp sex reassignment/ or exp male to female transsexual/ or exp "sexual and gender minority"/ or exp gender dysphoria/  OR  (transgender* or trans-gender* or "gender divers*" or nonbinary or non-binary or "gender nonconforming" or "gender non-conforming" or "gender identity" or “gender incongruen*” or "trans m?n" or "trans wom?n" or "assigned female at birth" or AFAB or "assigned male at birth" or "sex assigned at birth" or AMAB or transsexual* or genderfluid* or gender-fluid* or genderqueer* or gender-queer* or agender*).mp. |
| AND | |
| **Phenomena of Interest** | exp personal experience/ or exp attitude/ or exp attitude to health/ or exp patient attitude/ or exp emotion/ or exp health belief/ OR (experience* or perception* or perceiv* or attitude* or perspective* or feeling* or thought* or viewpoint* or opinion* or belief* or barrier*).mp. |
| AND | |
| **Context**  (geographical location) | exp United Kingdom/ or exp national health service/  OR  (national health service* or nhs*).mp.  OR  (gb or "g.b." or britain* or (british* not "british columbia") or uk or "u.k." or united kingdom* or (england* not "new england") or northern ireland* or northern irish* or scotland* or scottish* or ((wales* or "south wales*") not "new south wales") or welsh*).mp.  OR  (english not ((published or publication* or translat* or written or language* or speak* or literature or citation*) adj5 english)).mp.  OR  (bath or "bath's" or (birmingham not alabama*) or ("birmingham's" not alabama*) or bradford or "bradford's" or brighton or "brighton's" or bristol or "bristol's" or carlisle or "carlisle's" or (cambridge not (massachusetts* or boston* or harvard*)) or ("cambridge's" not (massachusetts* or boston* or harvard*)) or (canterbury not zealand*) or ("canterbury's" not zealand*) or chelmsford or "chelmsford's" or chester or "chester's" or chichester or "chichester's" or coventry or "coventry's" or derby or "derby's" or (durham not (carolina* or nc)) or ("durham's" not (carolina* or nc)) or ely or "ely's" or exeter or "exeter's" or gloucester or "gloucester's" or hereford or "hereford's" or hull or "hull's" or lancaster or "lancaster's" or leeds* or leicester or "leicester's" or (lincoln not nebraska*) or ("lincoln's" not nebraska*) or (liverpool not (new south wales* or nsw)) or ("liverpool's" not (new south wales* or nsw)) or ((london not (ontario* or ont or toronto*)) or (("london's" not (ontario or ont or toronto*)) or manchester or "manchester's" or (newcastle not (new south wales* or nsw)) or ("newcastle's" not (new south wales* or nsw)) or norwich or "norwich's" or nottingham or "nottingham's" or oxford or "oxford's" or peterborough or "peterborough's" or plymouth or "plymouth's" or portsmouth or "portsmouth's" or preston or "preston's" or ripon or "ripon's" or salford or "salford's" or salisbury or "salisbury's" or sheffield or "sheffield's" or southampton or "southampton's" or st albans or stoke or "stoke's" or sunderland or "sunderland's" or truro or "truro's" or wakefield or "wakefield's" or wells or westminster or "westminster's" or winchester or "winchester's" or wolverhampton or "wolverhampton's" or (worcester not (massachusetts* or boston* or harvard*)) or ("worcester's" not (massachusetts* or boston* or harvard*)) or (york not ("new york" or ny or ontario* or ont or toronto*)) or ("york's" not ("new york" or ny or ontario* or ont or toronto*))))).ti,ab.  OR  (bangor or "bangor's" or cardiff or "cardiff's" or newport or "newport's" or st asaph or "st asaph's" or st davids or swansea or "swansea's").ti,ab.  OR  (aberdeen or "aberdeen's" or dundee or "dundee's" or edinburgh or "edinburgh's" or glasgow or "glasgow's" or inverness or (perth not australia*) or ("perth's" not australia*) or stirling or "stirling's").ti,ab.  OR  (armagh or "armagh's" or belfast or "belfast's" or lisburn or "lisburn's" or londonderry or "londonderry's" or derry or "derry's" or newry or "newry's").ti,ab.  above NOT  (exp "arctic and antarctic"/ or exp oceanic regions/ or exp western hemisphere/ or exp africa/ or exp asia/ or exp "australia and new zealand"/) not (exp united kingdom/ or europe/) |
| AND | |
| **Publication Date** | 2019 - current |
| AND | |
| **Additional Limits** | exclude books, chapter, conference abstract, conference paper, conference review, editorial, erratum, letter, note, tombstone |

| **Database** | MEDLINE® ALL |
| --- | --- |
| **Coverage** | 1946 to October 29, 2024 |
| **Platform** | Ovid |
|  | |
| **PICo** | **MEDLINE Search Terms** |
| **Population** | exp Transgender Persons/ or exp Health Services for Transgender Persons/ or exp Transsexualism/ or exp Gender Identity/ or exp "Sexual and Gender Minorities"/ or exp Gender-Nonconforming Persons/ or exp Gender Dysphoria/  OR  (transgender* or trans-gender* or "gender divers*" or nonbinary or non-binary or "gender nonconforming" or "gender non-conforming" or "gender identity" or “gender incongruen*” or "trans m?n" or "trans wom?n" or "assigned female at birth" or AFAB or "assigned male at birth" or "sex assigned at birth" or AMAB or transsexual* or genderfluid* or gender-fluid* or genderqueer* or gender-queer* or agender*).mp. |
| AND | |
| **Phenomena of Interest** | exp Attitude to Health/ or exp Attitude/ or exp Emotions/  OR (experience* or perception* or perceiv* or attitude* or perspective* or feeling* or thought* or viewpoint* or opinion* or belief* or barrier*).mp. |
| AND | |
| **Context**  (geographical location) | exp United Kingdom/  OR  (national health service* or nhs*).mp.  OR  (gb or "g.b." or britain* or (british* not "british columbia") or uk or "u.k." or united kingdom* or (england* not "new england") or northern ireland* or northern irish* or scotland* or scottish* or ((wales* or "south wales*") not "new south wales") or welsh*).mp.  OR  (english not ((published or publication* or translat* or written or language* or speak* or literature or citation*) adj5 english)).mp.  OR  (bath or "bath's" or (birmingham not alabama*) or ("birmingham's" not alabama*) or bradford or "bradford's" or brighton or "brighton's" or bristol or "bristol's" or carlisle or "carlisle's" or (cambridge not (massachusetts* or boston* or harvard*)) or ("cambridge's" not (massachusetts* or boston* or harvard*)) or (canterbury not zealand*) or ("canterbury's" not zealand*) or chelmsford or "chelmsford's" or chester or "chester's" or chichester or "chichester's" or coventry or "coventry's" or derby or "derby's" or (durham not (carolina* or nc)) or ("durham's" not (carolina* or nc)) or ely or "ely's" or exeter or "exeter's" or gloucester or "gloucester's" or hereford or "hereford's" or hull or "hull's" or lancaster or "lancaster's" or leeds* or leicester or "leicester's" or (lincoln not nebraska*) or ("lincoln's" not nebraska*) or (liverpool not (new south wales* or nsw)) or ("liverpool's" not (new south wales* or nsw)) or ((london not (ontario* or ont or toronto*)) or (("london's" not (ontario or ont or toronto*)) or manchester or "manchester's" or (newcastle not (new south wales* or nsw)) or ("newcastle's" not (new south wales* or nsw)) or norwich or "norwich's" or nottingham or "nottingham's" or oxford or "oxford's" or peterborough or "peterborough's" or plymouth or "plymouth's" or portsmouth or "portsmouth's" or preston or "preston's" or ripon or "ripon's" or salford or "salford's" or salisbury or "salisbury's" or sheffield or "sheffield's" or southampton or "southampton's" or st albans or stoke or "stoke's" or sunderland or "sunderland's" or truro or "truro's" or wakefield or "wakefield's" or wells or westminster or "westminster's" or winchester or "winchester's" or wolverhampton or "wolverhampton's" or (worcester not (massachusetts* or boston* or harvard*)) or ("worcester's" not (massachusetts* or boston* or harvard*)) or (york not ("new york" or ny or ontario* or ont or toronto*)) or ("york's" not ("new york" or ny or ontario* or ont or toronto*))))).ti,ab.  OR  (bangor or "bangor's" or cardiff or "cardiff's" or newport or "newport's" or st asaph or "st asaph's" or st davids or swansea or "swansea's").ti,ab.  OR  (aberdeen or "aberdeen's" or dundee or "dundee's" or edinburgh or "edinburgh's" or glasgow or "glasgow's" or inverness or (perth not australia*) or ("perth's" not australia*) or stirling or "stirling's").ti,ab.  OR  (armagh or "armagh's" or belfast or "belfast's" or lisburn or "lisburn's" or londonderry or "londonderry's" or derry or "derry's" or newry or "newry's").ti,ab.  above NOT  (exp africa/ or exp americas/ or exp antarctic regions/ or exp arctic regions/ or exp asia or exp oceania) not (exp great britain/ or europe/) |
| AND | |
| **Publication Date** | 2019 - current |

| **Database** | APA PsycINFO |
| --- | --- |
| **Coverage** | 1987 to October 2024 Week 4 |
| **Platform** | Ovid |
|  | |
| **PICo** | **PsycINFO Search Terms** |
| **Population** | exp "Transgender (Attitudes Toward)"/ or exp Transgender/ or exp Gender Nonbinary/ or exp Gender Nonconforming/ or exp Gender Identity/ or exp Transsexualism/ or exp Gender Dysphoria/  OR  (transgender* or trans-gender* or "gender divers*" or nonbinary or non-binary or "gender nonconforming" or "gender non-conforming" or "gender identity" or “gender incongruen*” or "trans m?n" or "trans wom?n" or "assigned female at birth" or AFAB or "assigned male at birth" or "sex assigned at birth" or AMAB or transsexual* or genderfluid* or gender-fluid* or genderqueer* or gender-queer* or agender*).mp. |
| AND | |
| **Phenomena of Interest** | exp Attitude Formation/ or exp Emotions/ or exp Client Attitudes/ or exp Attitudes/ or exp Life Experiences/ or exp Health Attitudes/  OR (experience* or perception* or perceiv* or attitude* or perspective* or feeling* or thought* or viewpoint* or opinion* or belief* or barrier*).mp. |
| AND | |
| **Context**  (healthcare setting) | exp Mental Health Services/ or exp Health Care Services/ or exp Health Care Delivery/ or exp Primary Health Care/ or exp Hospitals/ or exp Outpatient Treatment/ or exp Health Care Utilization/ or exp "Quality of Care"/ or exp Clinics/ or exp Pharmacy/ or exp Patients/ or exp Outpatients/ OR ("healthcare" or "health-care" or "medical care" or "health service*" or "primary care" or "primary healthcare" or "primary health care" or "GP" or "general practi*" or "secondary care" or "secondary healthcare" or "secondary health care" or "tertiary care" or "tertiary healthcare" or "tertiary health care" or "hospital*" or "hospital care" or "clinic" or "clinics" or "pharmacy" or "pharmacies" or "patient*" or "patient care" or "outpatient*" or "outpatient care").mp. |
| AND | |
| **Context**  (geographical location) | (gb or "g.b." or britain* or (british* not "british columbia") or uk or "u.k." or united kingdom* or (england* not "new england") or northern ireland* or northern irish* or scotland* or scottish* or ((wales or "south wales") not "new south wales") or welsh*).mp.  OR  (english not ((published or publication* or translat* or written or language* or speak* or literature or citation*) adj5 english)).mp.  OR  (national health service* or nhs*).mp.  OR  (bath or "bath's" or (birmingham not alabama*) or ("birmingham's" not alabama*) or bradford or "bradford's" or brighton or "brighton's" or bristol or "bristol's" or carlisle or "carlisle's" or (cambridge not (massachusetts* or boston* or harvard*)) or ("cambridge's" not (massachusetts* or boston* or harvard*)) or (canterbury not zealand*) or ("canterbury's" not zealand*) or chelmsford or "chelmsford's" or chester or "chester's" or chichester or "chichester's" or coventry or "coventry's" or derby or "derby's" or (durham not (carolina* or nc)) or ("durham's" not (carolina* or nc)) or ely or "ely's" or exeter or "exeter's" or gloucester or "gloucester's" or hereford or "hereford's" or hull or "hull's" or lancaster or "lancaster's" or leeds* or leicester or "leicester's" or (lincoln not nebraska*) or ("lincoln's" not nebraska*) or (liverpool not (new south wales* or nsw)) or ("liverpool's" not (new south wales* or nsw)) or ((london not (ontario* or ont or toronto*)) or (("london's" not (ontario or ont or toronto*)) or manchester or "manchester's" or (newcastle not (new south wales* or nsw)) or ("newcastle's" not (new south wales* or nsw)) or norwich or "norwich's" or nottingham or "nottingham's" or oxford or "oxford's" or peterborough or "peterborough's" or plymouth or "plymouth's" or portsmouth or "portsmouth's" or preston or "preston's" or ripon or "ripon's" or salford or "salford's" or salisbury or "salisbury's" or sheffield or "sheffield's" or southampton or "southampton's" or st albans or stoke or "stoke's" or sunderland or "sunderland's" or truro or "truro's" or wakefield or "wakefield's" or wells or westminster or "westminster's" or winchester or "winchester's" or wolverhampton or "wolverhampton's" or (worcester not (massachusetts* or boston* or harvard*)) or ("worcester's" not (massachusetts* or boston* or harvard*)) or (york not ("new york" or ny or ontario* or ont or toronto*)) or ("york's" not ("new york" or ny or ontario* or ont or toronto*))))).ti,ab.  OR  (bangor or "bangor's" or cardiff or "cardiff's" or newport or "newport's" or st asaph or "st asaph's" or st davids or swansea or "swansea's").ti,ab.  OR  (aberdeen or "aberdeen's" or dundee or "dundee's" or edinburgh or "edinburgh's" or glasgow or "glasgow's" or inverness or (perth not australia*) or ("perth's" not australia*) or stirling or "stirling's").ti,ab.  OR  (armagh or "armagh's" or belfast or "belfast's" or lisburn or "lisburn's" or londonderry or "londonderry's" or derry or "derry's" or newry or "newry's").ti,ab. |
| AND | |
| **Publication Date** | 2019 - current |

| **Database** | Web of Science Core Collection |
| --- | --- |
| **Coverage** | 1900 – present (October 29, 2024) |
| **Platform** | Clarivate |
|  | |
| **PICo** | **Web of Science Search Terms** |
| **Population** | TS=(transgender* or trans-gender* or "gender divers*" or nonbinary or non-binary or "gender nonconforming" or "gender non-conforming" or "gender identity" or “gender incongruen*” or "trans m?n" or "trans wom?n" or "assigned female at birth" or AFAB or "assigned male at birth" or "sex assigned at birth" or AMAB or transsexual* or genderfluid* or gender-fluid* or genderqueer* or gender-queer* or agender*) |
| AND | |
| **Phenomena of Interest** | TS=(experience* or perception* or perceiv* or attitude* or perspective* or feeling* or thought* or viewpoint* or opinion* or belief* or barrier*) |
| AND | |
| **Context**  (healthcare setting) | TS=(healthcare or health-care or "health care" or "medical care" or "health service*" or "primary care" or "primary healthcare" or "primary health care" or GP or "general practi*" or "secondary care" or "secondary healthcare" or "secondary health care" or "tertiary care" or "tertiary healthcare" or "tertiary health care" or hospital* or "hospital care" or clinic or clinics or pharmacy or pharmacies or patient* or "patient care" or outpatient* or "outpatient care") |
| AND | |
| **Context**  (geographical location) | TS=(gb or "g.b." or britain* or (british* not "british columbia") or uk or "u.k." or "united kingdom*" or (england* not "new england") or english* or "northern ireland*" or "northern irish*" or scotland* or scottish* or ((wales* or "south wales*") not "new south wales") or welsh*) OR TS=(“national health service*” or nhs*) OR TI/AB=("bath" or ("birmingham" not "alabama*") or "bradford" or "brighton" or "bristol" or "carlisle" or ("cambridge" not ("massachusetts*" or "boston*" or "harvard*")) or ("canterbury" not "zealand*") or "chelmsford" or "chester" or "chichester" or "coventry" or "derby" or ("durham" not ("carolina*" or "nc")) or "ely" or "exeter " or "gloucester" or "hereford" or "hull" or "lancaster" or "leeds" or "leicester" or ("lincoln" not "nebraska*") or ("liverpool" not ("new south wales*" or "nsw")) or (("london" not ("ontario*" or "ont" or "toronto*")) or "manchester" or ("newcastle" not ("new south wales*" or "nsw")) or "norwich" or "nottingham" or "oxford" or "peterborough" or "plymouth" or "portsmouth" or "preston" or "ripon" or "salford" or "salisbury" or "sheffield" or "southampton" or "st albans" or "stoke" or "sunderland" or "truro" or "wakefield" or "wells" or "westminster" or "winchester" or "wolverhampton" or ("worcester" not ("massachusetts*" or "boston*" or "harvard*")) or ("york" not ("new york" or "ny" or "ontario*" or "ont" or "toronto*")) or “bangor” or “cardiff” or “newport” or “st asaph” or “st davids” or “swansea” or “aberdeen” or “dundee” or “edinburgh” or “glasgow” or “inverness” or (“perth” not “australia*”) or “stirling” or “armagh” or “belfast” or “lisburn” or “londonderry” or “derry” or “newry”) ) |
| AND | |
| **Publication Date** | 2019-01-01 – 2024-10-29 |
