# Supplementary material for: Healthcare experiences of transgender and gender-diverse people in the UK: a scoping review
Source: BMJ Open. 2026 May 4;16(5):e106519. doi: 10.1136/bmjopen-2025-106519 (PMC13141246; doi:10.1136/bmjopen-2025-106519)
Supplement: online supplemental file 3 [file bmjopen-16-5-s003.docx]

| **Author, Year** | **Objectives** | **Methods** | **Sample** | **Location** | **Healthcare Setting(s)** | **Community Involvement** | **Funding** |
| --- | --- | --- | --- | --- | --- | --- | --- |
| Berner et al. (2021)  29 | TGD healthcare | Mixed-Methods - survey *(qualitative & quantitative)* | TGD only (n=137)  - trans men & non-binary people | UK only  - no further information | - GP  - Screening services | LGBT+ organisation consultation /involvement | No funding associated |
| Bower-Brown (2022)  30 | TGD experiences  - not healthcare-specific | Qualitative  - interviews | TGD only (n=13)  - TGD parents or expectant parents | UK only  - no further information | - Fertility services  - Unclear | LGBT+ research team | Wellcome Trust |
| Bower-Brown & Zadeh (2021)  31 | TGD experiences  - not healthcare-specific | Qualitative  - interviews | TGD only (n=11)  - TGD parents | UK only  - England  *(urban & rural settings)* | - Fertility services  - Perinatal services | None mentioned | Charitable foundation (health focus) |
| Brenisin et al. (2022)  32 | Healthcare experiences | Qualitative  - interviews | Total sample (n=11)  - TGD participants (n=2)  - mental health inpatients | UK only  - no further information | - Mental health services | None mentioned | NHS England |
| Braybrook et al. (2022)  33 | LGBT+ healthcare experiences | Qualitative  - interviews | LGBT+ sample (n=74)  - TGD participants (n=8)  - people facing serious illness | UK only - England | - Hospital  - Unclear | None mentioned | NIHR |
| Bruce et al. (2023)  34 | TGD healthcare experiences | Qualitative  - interviews | TGD only  - UK participants (n=3)  - total sample (n=17)  - autistic adults seeking gender-affirming care | Multi-National  - England  *(Cornwall; Newcastle; Teeside)* | - Gender services  - GP  - Private healthcare  - Unclear | TGD research team | No funding |
| Carlile (2020)  35 | TGD healthcare experiences | Qualitative  - written responses following discussion workshops | TGD young people & their parents  - number of TGD participants unclear  - total sample (n=65)  *(from 27 families “around half the respondents were adults … the remainder were young people”)* | UK only  - England | - Gender services  - GP  - Unclear | LGBT+ research team | No information |
| Carlile et al. (2021)  36 | TGD healthcare experiences | Qualitative  - interviews | TGD children & their parents  - TGD participants (n=14) - parent sample (n=13)  - children & young people | UK only  - England | - Gender services  - GP  - Mental health services | None mentioned | No information |
| Coleman‑Smith et al. (2020)  37 | TGD experiences  - not healthcare-specific | Qualitative  - interviews | TGD only (n=10)  - autistic adults | UK only  - no further information | - gender services | None mentioned | No information |
| Connolly et al. (2024)  38 | TGD healthcare experiences | Mixed-Methods  - survey *(qualitative & quantitative)* | TGD only (n=565)  - respondents to Open Question 1 (n=65)  - respondents to Open Question 2 (n=111)  - current or former alcohol users | UK only  - UK wide | - GP  - Mental health services  - Unclear | - TGD-led charity consultation/ involvement  - LGBT+ organisation consultation/involvement | Academic institution |
| Cooper et al. (2022)  39 | TGD experiences  - not healthcare-specific | Qualitative  interviews | TGD only (n=21)  - autistic adults | UK only  - no further information | - Gender services  - GP  - Unclear | TGD consultation | NIHR |
| Dimova et al. (2022)  40 | LGBT+ healthcare experiences | Qualitative  - interviews | LGBT+ sample (n=14)  - TGD participants (n=4) | UK only - Scotland | - GP | None mentioned | Public health advocacy organisation |
| Glackin et al.  2024  41 | TGD healthcare experiences | Qualitative  - interviews  - survey | TGD only (n=12)  - autistic adults | UK only  - no further information | - Mental health services  - Unclear | None mentioned | No funding |
| Grant et al. (2021)  42 | TGD healthcare experiences | Qualitative  - interviews | TGD only (n=9)  - trans adults | UK only  - London, England | - Gender services  - Mental health services  - Unclear | TGD consultation | No information |
| Greenfield & Darwin (2024)  43 | LGBT+ healthcare experiences  - COVID-19 | Qualitative  - survey | LGBT+ sample (n=76)  - TGD participants (n=2)  - parents | UK only  - no further information | - Perinatal services | None mentioned | ESRC; NIHR |
| Griffin et al. (2023)  44 | LGBT+ health | Qualitative - focus groups | LGBT+ sample (n=20)  - TGD participants (n=14)  - children & young people | UK only  - North-East, England | - Gender services  - GP  - Mental health services | LGBT+ organisation consultation  /involvement | NIHR |
| Habicht et al. (2024)  45 | Healthcare | Mixed-Methods  - analysis of qualitative feedback responses  - referral statistics | Total sample (n=129,400)  - TGD sample (n=640)  Participants providing qualitative feedback (n=42,332)  - no information on TGD proportion of this sample | UK only - England | - Mental health services | None mentioned | No information |
| Hafford-Letchfield et al. (2022)  46 | LGBT+ experiences  - COVID-19 | Qualitative  - interviews | LGBT+ sample (n=17)  - TGD participants (n=5)  - older adults | UK only  - no further information | - Gender services | None mentioned | No information |
| Harrison et al. (2020)  47 | TGD healthcare experiences | Qualitative  - interviews | TGD only (n=8)  - trans adults | UK only  - East Midlands, England | - Gender services  - GP  - Private healthcare | None mentioned | No information |
| Haworth et al. (2023)  48 | LGBT+ experiences  - COVID-19 | Qualitative  - interviews | LGBT+ sample (n=17)  - TGD participants (n=8) | International  - England  - Scotland | - Gender services | LGBT+ research team | ESRC |
| Hibbert et al. (2019)  49 | TGD healthcare experiences | Quantitative  - survey  TGD subgroup analysis | TGD only (n=500) | UK only  - no further information | - Sexual health services  - Unclear | LGBT+ organisation consultation /involvement | Academic institution |
| Holti et al. (2024)  50 | TGD healthcare experiences | Mixed-Methods  - interviews  - focus groups  - survey *(quantitative)* – not reported | TGD only (n=110)  - recruited from survey respondents (n=2056)  - older people  - low income and/or low education  - chronic illness or disabled  - living in rural areas  - Black people & People of Colour  - living with mental health conditions | UK only  - no further information | - Gender services  - GP  - Private healthcare  - Mental health services  - Sexual health services  - Hospital  - Screening services  - Unclear | - TGD research team  - TGD-led charity consultation/ involvement  - TGD consultation | HSDR; NIHR |
| Hord & Medcalf (2022)  51 | TGD healthcare experiences | Qualitative  - surveys  - workshops | TGD only (n=348)  - general survey (n=309)  - workshops (n=61) - trans POC survey (n=39) | UK only - England | - Gender services  - GP  - Private healthcare  - Mental health services  - Sexual health services  - Fertility services  - Hospital  - Screening services  - Unclear | TGD consultation | National Community Lottery Fund |
| Horton (2022)  52 | TGD healthcare experiences | Qualitative  - interviews | TGD children & their parents  - TGD participants (n=10) - parent sample (n=30) | UK only  - England  - Scotland  - Wales | - Gender services | TGD research team | UK university |
| Horton (2024)  52 | TGD healthcare experiences | Qualitative  - interviews | TGD children & their parents  - TGD participants (n=10) - parent sample (n=30) | UK only  - no further information | - Gender services | TGD research team | Unclear |
| Jackson et al. (2023)  54 | TGD healthcare experiences | Qualitative  - interviews | TGD only (n=7)  - chest/breastfeeding parents | UK only  - England (South city; East Midlands city)  - Scotland (city) - Wales (city & town) | - Gender services  - Perinatal services | TGD consultation | No funding |
| Jones et al. (2023)  55 | TGD experiences  - COVID-19  - not healthcare-specific | Mixed-Methods  - survey *(qualitative & quantitative)* | TGD only (n=161)  - young people | UK only  - no further information | - Gender services  - Mental health services  - Unclear | None mentioned | No funding |
| Kamran et al. (2023)  56 | TGD healthcare experiences | Qualitative  - focus groups | TGD patients & their clinicians  - TGD participants (n=14) | UK only  - no further information | - Gender services  - GP | TGD consultation | NIHR |
| Lehmann et al. (2021)  57 | TGD healthcare experiences | Qualitative  - interviews  qualitative component of wider mixed-methods project | TGD only (n=40)  - gender service users | UK only  - Belfast, N. Ireland | - Gender services | TGD consultation | Public Health Agency Northern Ireland |
| Leven (2021)  58 | TGD healthcare experiences | Mixed-Methods  - focus groups  - interviews  - survey *(quantitative)* | TGD only (n=602)  - survey (n=521)  - groups/interviews (n=81) | UK only - Greater Glasgow & Clyde; Lothian, Scotland | - Gender services  - GP  - Mental health services  - Hospital  - Private healthcare  - Screening services  - Gynaecology services  - Setting unclear | None mentioned | Scottish government |
| LGBT Foundation (2020)  59 | LGBT+ focus  - COVID-19  - not healthcare-specific | Mixed-Methods  - survey *(qualitative & quantitative)* | LGBT+ sample (n=555)  - TGD sample size not stated | UK only  - no further information | - Gender services  - GP  - Unclear | LGBT+ organisation consultation/involvement | LGBT charity |
| LGBT Foundation  (2022)  60 | TGD healthcare experiences | Mixed-Methods - interviews  - survey *(qualitative & quantitative)* | TGD only (n=125)  - survey (n=121)  - interviews (n=4)  - TGD parents or expectant parents  - trans men & non-binary people | UK only  - no further information | - GP  - Fertility services  - Perinatal services | LGBT+ organisation consultation/involvement | Health & Wellbeing Alliance |
| McKay et al. (2022)  61 | TGD healthcare experiences | Qualitative  - interviews | TGD young people & their parents  - TGD participants (n=39)  - children & young people on waiting lists for gender services | UK only  - England  - Wales | - Gender services | TGD consultation | NIHR |
| Millet et al. (2024)  62 | LGBT+ healthcare experiences | Qualitative  - interviews | LGBT+ patients, their partners & their clinicians  - TGD patient participants (n=1)  - patients with uterine cancer | UK only  - no further information | - Gynaecology services | LGBT+ organisation consultation /involvement | Academic institution |
| Mills et al. (2023)  63 | TGD healthcare experiences | Qualitative  - interviews  - focus groups | TGD only (n=12)  - transmasculine people | UK only  - England  - Scotland | - Gender services  - GP  - Mental health services  - Unclear | TGD research team | No funding |
| Munday (2022)  64 | TGD experiences  - not healthcare-specific | Qualitative  - interviews | TGD only  - UK participants (n=4)  - total sample (n=15) | Multi-National  - UK locations not specified | - Unclear | TGD research team | No information |
| Palich et al. (2024)  65 | LGBT+ healthcare experiences | Qualitative  - interviews  sub-study with participants of the SELPHI RCT | LGBT+ sample (n=15)  - TGD participants (n=4)  - sexual assault survivors | UK only - England  - Wales | - Unclear | None mentioned | NIHR |
| Pipkin & Clarke (2024)  66 | TGD healthcare experiences | Qualitative  - focus groups | TGD only (n=6)  - trans People of Colour | UK only  - no further information | - Gender services | - TGD consultation  - TGD research team | No funding |
| Riggs et al. (2021)  67 | TGD healthcare experiences | Qualitative  - interviews | TGD only  - UK participants (n=14)  - Total sample (n=51)  - trans men, transmasculine & non-binary people negotiating conception | International  - UK locations not specified | - general practice  - fertility services | TGD research team | ESRC |
| Shepherd & Hanckel (2020)  68 | TGD healthcare experiences | Mixed-Methods  - interviews  - analysis of NHS docs | TGD only (n=59)  - adults transitioning in healthcare | UK only  - England (urban & regional areas) | - Gender services  - GP  - Private healthcare | TGD research team | No information |
| Spence et al. (2024)  69 | Healthcare experiences | Qualitative  - interviews | Total sample (n=100)  - TGD participants (n=10) | UK only  - no further information | - Sexual health services  - Pharmacy services | None mentioned | NIHR |
| Squires et al. (2024)  70 | TGD healthcare experiences | Qualitative  - interviews | TGD only (n=10) | UK only  - no further information | - Gender services  - GP  - Private healthcare | LGBT+ organisation consultation /involvement | Academic institution |
| Steele et al. (2020)  71 | TGD healthcare experiences | QUANTITATIVE  - survey | TGD only (n=53)  - sex workers | UK only  - no further information | - GP  - Unclear | None mentioned | ECDC; Wellcome Trust; ERC |
| Suhomlinova et al.  (2022)  72 | TGD experiences  - COVID-19  - not healthcare-specific | Qualitative  - letter writing | TGD only (n=15)  - trans women & non-binary people  - prisoners | UK only  - England  - Wales | - Gender services  - GP  - Prison healthcare | None mentioned | Academic institution |
| Tasker & Gato (2020)  73 | TGD focus  - not healthcare-specific | Qualitative  - focus groups | TGD only (n=11) | UK only  - England  *(“in and around the London area”)* | - Gender services  - Fertility services | TGD-led charity consultation/ involvement | Academic institution |
| Taylor et al. (2019)  74  (~~42)~~ | TGD healthcare experiences | Qualitative  - focus groups  *(part of a service evaluation at an NHS gender identity clinic)* | TGD only (n=8) | UK only  - no further information | - Gender services | None mentioned | No information |
| Toze et al. (2023)  75 | TGD focus  - COVID-19 | Mixed-Methods  - survey *(qualitative & quantitative)*    TGD subgroup analysis | TGD only (n=38)  - older adults | UK only  - no further information | - Gender services  - Unclear | None mentioned | No information |
| TransActual (2021)  76 | TGD healthcare experiences | Mixed-Methods  - survey *(qualitative & quantitative)* | TGD only (n=697) | UK only  - no further information | - Gender services  - GP  - Private healthcare  - Screening services  - Unclear | TGD-led charity consultation/ involvement | TGD charity |
| Williams et al. (2024)  77 | LGBT+ healthcare experiences | Qualitative  - interviews | LGBT+ sample (n=8)  - TGD participants (n=4)  - young Black & mixed race people living with physical health conditions | UK only  - England | - Unclear | LGBT+ research team | No funding |
| Willis et al. (2020)  78 | TGD healthcare experiences | Qualitative  - interviews | TGD only (n=22)  - older adults | UK only  - England  - Wales | - Gender services  - GP  - Private healthcare  - Mental health services  - Unclear | None mentioned | Charitable foundation |
| Willis et al. (2021)  79 | TGD focus  - not healthcare-specific | Qualitative  interviews | TGD only (n=22)  - older adults | UK only  - Wales | - Gender services  - Mental health services | TGD consultation | Charitable Foundation |
| Witney et al. (2024)  80 | TGD healthcare experiences | Qualitative  - interviews  - focus groups | TGD only (n=59) | UK only  - no further information | - Gender services  - Sexual health services  - Unclear | None mentioned | No information |
| Witzel et al. (2021)  81 | TGD healthcare experiences | Mixed-Methods  - interviews  - abridged RCT results reported  sub-study with participants of the SELPHI RCT | TGD only (n=20) | UK only  - England  - Wales | - Sexual health services | TGD research team | NIHR; Medical Research Council |
| Wright et al. (2021)  82 | TGD healthcare experiences | Qualitative  - interviews  sub-study with participants of the SELPHI RCT | TGD only (n=20) | UK only  - England  - Wales  75% of participants lived in the south of England (south-east, south-west, east, and London) | - Gender services  - GP  - Private healthcare  - Mental health services  - Hospital  - Unclear | None mentioned | NIHR |
