# Supplementary material for: Healthcare experiences of transgender and gender-diverse people in the UK: a scoping review
Source: BMJ Open. 2026 May 4;16(5):e106519. doi: 10.1136/bmjopen-2025-106519 (PMC13141246; doi:10.1136/bmjopen-2025-106519)
Supplement: Supplementary file 4 [file bmjopen-2025-106519.reviewer_comments.pdf]

PEER REVIEW HISTORY

BMJ Open publishes all reviews undertaken for accepted manuscripts. Reviewers are asked to complete a checklist review form and are provided with free text boxes to elaborate on their assessment. These free text comments are reproduced below.

ARTICLE DETAILS

Title (Provisional)

Healthcare Experiences of transgender & gender-diverse people in the UK: a scoping review

Authors

Davidson, Lauren; Moore, Aiden; Grove, Amy; Gauly, Julia

VERSION 1 - REVIEW

**Reviewer**	**1**
**Name**	**Roth, Stephanie**
**Affiliation**	**ChristianaCare**
**Date**	**28-Jul-2025**
**COI**	**Owner of a personal business, providing education/consulting on reviews.**

At first glance, this looked like a high-quality review, and it mentioned PRISMA and PRISMA-SCR was followed for the reporting, but below the surface, there are some areas within the methodology that are questionable and weakens the overall review. Despite consulting the guidance for reporting, I found the main issues with the review are in the reporting.

I believe there was a good faith attempt to make this a comprehensive and systematic search, but there are some reporting flaws.

Since the search is the heart of the findings, I find it problematic that the reporting of the search is missing some key details.

1.) Search-not following PRISMA-S

It looks like you have consulted with a librarian to create a decent search, but the documentation doesn't meet PRISMA-S (missing platform names, no date justifications, no database years, use of database limits, search not peer-reviewed using PRESS by another librarian). It is not clear who conducted the search. Since it's a scoping review, do you have a clear reason, supporting not searching the grey literature. It's mentioned as a limitation briefly, but it seems like it's still not adequately addressed the reasons for not including it.

2.) Strengths and Limitations reported briefly with the abstract but not elaborated on in the paper, the discussion section was missing this information, and it didn't address bias.

3.) Was screening blinded in Rayyan? How were duplicates removed-in Rayyan?

4.) Seems more like a rapid scoping review/rapid review to take a shortcut in the data extraction phase-using a single author to extract data and a second to only extract 15%.

There was no explanation for why the other stages had two reviewers, but the data extraction needed to be processed more rapidly by mainly one.

These methodological issues should be addressed before it will be acceptable to the readership.

Thank you for the opportunity to peer review your scoping review!

**Reviewer**	**2**
**Name**	**Ziegler, Erin**
**Affiliation**	**Ryerson University**
**Date**	**19-Aug-2025**
**COI**	**None**

Thank you for letting me reviewing this important work.

p4 line 6 - the correct term is term - is sex assign at birth. it is also no longer academically acceptable to use the "at odds with the perceived norms of their society" as this is concerned very discriminatory language and actually has nothing to do with how one identifies. You are correct at the beginning TDG is an inclusive umbrella term for those whose gender identity does not align with the sex assigned at birth

Pg6 - information sources and search - why was nursing database (CINHAL, EMCare) not included in your comprehensive coverage?

pg7 line 48-49 - "Forty-five records, reporting 43 studies, were eligible for inclusion.

An additional 12 records, reporting 11 studies" = this is confusing. 45 records, but reporting 43 studies? where are the other 2? Same with the next line? Duplicate? this is unclear and needs to be clarified. Are they the two combined results mentioned next?

I think rephrasing to 45 manuscripts were eligible for inclusion. Then describe that you had 2 with overlapping data. Then report the 12 additional manuscripts from reference list screening. If I have interpreted this correctly this will make it easier to understand.

pg8 - line 14 - what does " although many of these (n=25) did not further specify participant location" mean? that they may be also outside the UK? or unclear where in the UK?

p8-line 18 - Transition-related care settings - does this mean care settings that provider gender-affirming care? In North America transition care settings is a term that is used for patients moving from acute to long-term care so this usage here is confusing for international readers. Could you consider changing it - perhaps to gender-affirming care settings?

pg9 - line 5-6 - adaptions to accommodate autism - this is important but feels out of place in this sentence. Could it have its own sentence?

p9 - line 17 - what is the definition of "good care" what does that mean?

Discussion - this is very short and lacking any integration with literature. It should also highlight the "so what" and "what next". Some of what is in your conclusion can be moved to the discussion

---

VERSION 1 - AUTHOR RESPONSE

**Reviewer 1: Ms. Stephanhie Roth, ChristianaCare**
**At first glance, this looked like a high-quality review, and it mentioned PRISMA and PRISMA-SCR was followed for the reporting, but below the surface, there are some areas within the methodology that are questionable and weakens the overall review. Despite consulting the guidance for reporting, I found the main issues with the review are in the reporting.**<br><br>**I believe there was a good faith attempt to make this a comprehensive and systematic search, but there are some reporting flaws.**<br><br>**Since the search is the heart of the findings, I find it problematic that the reporting of the search is missing some key details.**
**Response:** Many thanks for taking your time to review our manuscript – we very much appreciate and have addressed all your comments as outlined below.
**1.) Search-not following PRISMA-S**<br>**It looks like you have consulted with a librarian to create a decent search, but the documentation doesn't meet PRISMA-S (missing platform names, no date justifications, no database years, use of database limits, search not peer-reviewed using PRESS by another librarian). It is not clear who conducted the search. Since it's a scoping review, do you have a clear reason, supporting not searching the grey literature. It's mentioned as a limitation briefly, but it seems like it's still not adequately addressed the reasons for not including it.**
**Response:**<br>We have now added the missing platform names to Supplementary File 1 (Search Strategies), along with database coverage years. The justification for date restrictions, previously in ‘Eligibility Criteria,’ is now included under ‘Information Sources & Search’ along with details and justification for the use of database limits. The initial search strategy was developed by the first and last authors and refined in consultation with a librarian, who reviewed the strategy and provided feedback to enhance its accuracy and comprehensiveness. This has now been reported. Although

the PRESS checklist was not formally used, this process ensured that key concepts, terms, and search syntax were appropriately applied. We have also clarified that the search of all databases was conducted by the first author on 29 October 2024. The rationale for not searching the grey literature is detailed under ‘Limitations’ in the Discussion.

Revision:

"*Information sources & search*

Database selection was carefully considered in consultation with a specialist librarian to provide comprehensive coverage of clinical (Embase, MEDLINE), psychological (PsycINFO), and multidisciplinary (Web of Science) research. **Initial search strategies were developed by the first and last author,** informed by the research questions and previous reviews conducted in this field (10, 12, 15), **and subsequently reviewed by a specialist librarian to ensure that key concepts, terms, and search syntax were appropriately applied.**

All database searches were conducted by the first author on 29 October 2024. Search terms were adapted for each database, combining relevant free-text and Medical Subject Heading (MeSH) terms using the Boolean operators (AND), (OR), and (NOT). Truncation techniques were utilised to capture variations in spelling and enhance retrieval. **Searches were limited to records published from 1 January 2019 to the most recent records available at the time of searching. This decision was informed by the publication of the eleventh revision of the International Classification of Diseases (ICD-11) in May 2019, which reclassified terms related to gender diversity (25). In ICD-10, the terms "transsexualism" and "gender identity disorders" are listed within "Disorders of adult personality and behaviour" (26). These terms do not appear in ICD-11, which introduces "gender incongruence" within a new category, "Conditions related to sexual health", and extends recognition to the experiences of children and adolescents (25). The updated classification marks a significant shift in the medical understanding of trans and gender-diverse identities.** Geographical search terms were informed by NICE UK filters to optimise identification of studies conducted in the UK (27). **Additional publication type limits were applied where available to align with the inclusion of primary research studies only.** Full search strategies for all databases are provided in Supplementary File 1. Reference lists of included studies were subsequently searched to identify further relevant literature. **No formal grey literature search was conducted.**"

2.) Strengths and Limitations reported briefly with the abstract but not elaborated on in the paper, the discussion section was missing this information, and it didn't address bias.

Response:

According to the PRISMA-ScR checklist, the Discussion section should focus on the limitations of the scoping review process. In line with this guidance, our manuscript previously included a 'Limitations' section within the Discussion, where we addressed methodological limitations and reviewer positionality. We have now more explicitly highlighted potential bias. In keeping with BMJ Open recommendations, we

have also highlighted the strengths of the review (e.g., first of its kind, geographic focus) in the Discussion within the ‘Conclusions’ subsection.

Revision:

Limitations

“**Grey literature was included through reference list screening. However, as no formal grey literature search was conducted due to time and resource constraints, insights from non-academic and advocacy sources may be underrepresented. While** the selected databases covered multiple disciplines, searching additional databases and hand-searching key journals could have enhanced the comprehensiveness of the review. Evolving terminology around TGD topics also poses challenges in identifying all relevant literature. The review team included a queer genderqueer person, a gay cisgender man, and two heterosexual cisgender women. We reflected on our individual and collective positionalities throughout, **with consideration of how this could influence the interpretation and synthesis of findings. We did not formally assess publication bias or study quality, which may limit the representativeness of our findings.**”

**3.) Was screening blinded in Rayyan? How were duplicates removed-in Rayyan?**
**Response:**<br>Articles were deduplicated using Rayyan’s standard (non-AI) duplicate detection. Blinding was enabled to prevent reviewers seeing each other’s decisions during both title/abstract and full-text screening stages. Blinding was lifted after all articles had been independently reviewed to allow identification of discrepancies. The ‘Selection of sources of evidence’ section has been revised to include this information.<br><br>**Revision:**<br>“Articles identified through database searches were imported into the web-based systematic review software, Rayyan (28), where **duplicates were detected, screened and removed by one reviewer using Rayyan's standard (non-AI) duplicate detection**. Titles and abstracts of the remaining records were independently screened by two reviewers against the eligibility criteria, with discrepancies resolved through discussion or screening by a third reviewer. Full texts of potentially relevant articles were then retrieved and independently screened by two reviewers, with reasons for exclusion recorded. Discrepancies were resolved through discussion or consultation with a third reviewer. **Reviewers were blinded to each other’s decisions during both independent screening stages; blinding was removed to identify and resolve discrepancies.**”
**4.) Seems more like a rapid scoping review/rapid review to take a shortcut in the data extraction phase-using a single author to extract data and a second to only extract 15%. There was no explanation for why the other stages had two reviewers, but the data extraction needed to be processed more rapidly by mainly one.**
**Response:**

Thank you for this comment. Data from 8 of the 52 included studies were independently extracted by the last author to check for consistency. As no disagreements arose during this independent extraction, all remaining studies were subsequently checked and revised by a second reviewer rather than undergoing full independent extraction. This ensured accuracy while streamlining the process. The 'Data charting' section has been revised to include this information.

Revision:
"A data charting form was developed based on the research questions, discussion within the review team, and the Arksey and O'Malley framework (23). **During the early stages of data charting, additional relevant data items were identified, specifically community involvement in study design, prompting a revision of the charting form. Data extraction was then completed by the primary reviewer using the finalised form.** Table 2 provides an overview of the extracted data items.

A representative subset of eight (15%) of the included studies was selected to reflect a range of study designs, methods, populations, and sample sizes. This subset was independently charted by a second reviewer to assess consistency and accuracy. As no disagreements arose during this independent extraction, all remaining studies were subsequently checked and revised by a second reviewer rather than undergoing full independent extraction. This ensured accuracy while streamlining the process. A final review of the dataset was conducted by the primary reviewer prior to synthesis."

These methodological issues should be addressed before it will be acceptable to the readership.

Thank you for the opportunity to peer review your scoping review!

Response: Thank you – we have addressed all methodological issues as outlined above to make our manuscript acceptable to the readership. Thank you again for your time to review our manuscript.

Reviewer: 2
Dr. Erin Ziegler, Ryerson University

Thank you for letting me reviewing this important work.

Response:
Many thanks for taking the time to review our manuscript and for your valuable comments – we very much appreciate it and have addressed all your point as outlined below.

p4 line 6 - the correct term is term - is sex assign at birth. it is also no longer academically acceptable to use the "at odds with the perceived norms of their society" as this is concerned very discriminatory language and actually has nothing to do with how one identifies. You are correct at the beginning TDG is an inclusive umbrella term for those whose gender identity does not align with the sex assigned at birth

Response:
Thank you. We agree and have revised the definition to use 'sex assigned at birth' and describe transgender and gender-diverse identities using identity-based terms.

Revision:
"Transgender and gender-diverse (TGD) is an inclusive umbrella term **encompassing people whose gender identity does not align with sex assigned at birth as well as those with gender identities or expressions that exist outside or beyond binary classifications** (1, 2)."

Pg6 - information sources and search - why was nursing database (CINHAL, EMCare) not included in your comprehensive coverage?

Response:
Database selection was informed by consultation with a specialist librarian and guided by previous reviews, none of which searched CINAHL or EMCARE, ensuring broad coverage of healthcare, clinical, psychological, and multidisciplinary literature. Medline and Embase, in particular, provide extensive indexing of nursing and allied health journals. To further capture relevant studies not included in the selected databases, supplementary strategies such as reference checking were employed. We acknowledge, however, that searching additional databases could have enhanced the comprehensiveness of the review, as noted in the 'Limitations' subsection of the Discussion.

pg7 line 48-49 - "Forty-five records, reporting 43 studies, were eligible for inclusion.
An additional 12 records, reporting 11 studies" = this is confusing. 45 records, but reporting 43 studies? where are the other 2? Same with the next line? Duplicate? this is unclear and needs to be clarified. Are they the two combined results mentioned next?
I think rephrasing to 45 manuscripts were eligible for inclusion. Then describe that you had 2 with overlapping data. Then report the 12 additional manuscripts from reference list screening. If I have interpreted this correctly this will make it easier to understand.

Response:
Thank you for highlighting this. The wording has been revised for clarity, with details of the total number of records retrieved from database searches and reference list screening retained in Figure 1 (PRISMA flow diagram) for transparency. Of the 54 studies that underwent data charting, two pairs were combined for the purposes of this review (as they were drawing on the same dataset), resulting in 52 studies included in the synthesis; this has also been clarified in the text.

Revision:
"Of 1,187 records identified through database searches, 76 full texts were retrieved following title and abstract screening. **Of these, 43 unique studies were eligible for inclusion. Reference list screening of included studies identified a further 11 unique studies eligible for inclusion. Data charting identified two results reporting overlapping and distinct data from the same participant sample (52, 53) and two results drawing from the same dataset (78, 79), which have been combined for the purposes of this review.** A further two results which drew from the same participant sample have been retained as separate studies due to their distinct objectives and analytic focus (81, 82). **Accounting for these combinations, a total**

**of 52 studies were included in the synthesis.** The selection process and reasons for exclusion following full-text screening are presented in Figure 1."
**pg8 - line 14 - what does " although many of these (n=25) did not further specify participant location" mean? that they may be also outside the UK? or unclear where in the UK?**
**Response:**<br>We agree that the original wording could be ambiguous. The sentence has been revised to clarify that in many of the UK-only studies, the precise location within the UK was not reported.<br><br>**Revision:**<br>"Almost all studies (n=48) involved UK-based participants only, although **25 of these did not report the specific location of participants within the UK**."
**p8-line 18 - Transition-related care settings - does this mean care settings that provider gender-affirming care? In North America transition care settings is a term that is used for patients moving from acute to long-term care so this usage here is confusing for international readers. Could you consider changing it - perhaps to gender-affirming care settings?**
**Response:**<br>We appreciate that the term "transition-related care" may be confusing for international readers. We purposefully did not use "gender-affirming care" to reflect that social support and affirmation should be provided in all healthcare settings, not only in services related to medical transition.<br>We therefore use "transition-related" to refer to care settings providing services specifically related to gender transition, including specialist gender services as well as transition-focused interactions within general practice and other healthcare settings. The first use of "transition-related care" in the Results section has been revised to clarify this and inform understanding of subsequent use of the term.<br><br>**Revision:**<br>"Transition-related care settings**, defined for the purpose of this review as services providing care specifically related to gender transition,** were most prevalent (n=33). These included specialist gender services accessed privately (n=10) or via NHS (National Health Service) pathways (n=32), as well as interactions within general practice (n=16) or mental health services (n=4)."
**pg9 - line 5-6 - adaptions to accommodate autism - this is important but feels out of place in this sentence. Could it have its own sentence?**
**Response:**<br>Thank you. As suggested, we have added a sentence expanding on adaptions to accommodate autism.<br><br>**Revision:**<br>"At gender clinic appointments, participants appreciated having time to discuss their concerns (37, 42, 50) **and** the thorough assessments provided (57). **Adaptations to accommodate autistic participants' communication and sensory needs were also valued (34, 39).**"

**p9 - line 17 - what is the definition of "good care" what does that mean?**
**Response:**<br>Thank you for this comment. We have revised the text to replace “good care” with “respectful and affirming care” to provide a clearer, more specific description of the type of care we are referring to.<br><br>**Revision:**<br>“In general healthcare settings, the proactive use of inclusive language was identified as a facilitator of **respectful and affirming** care.”
**Discussion - this is very short and lacking any integration with literature. It should also highlight the "so what" and "what next". Some of what is in your conclusion can be moved to the discussion**
**Response:**<br>Previously, the Discussion section was structured into three subsections (‘Summary of evidence’, ‘Limitations’, ‘Conclusions’) in line with PRISMA-ScR guidance for scoping reviews. We have added the subheading ‘Summary of evidence’ for clarity and readability. Integration with existing literature, implications (“so what”), and priorities for future research (“what next”) are addressed in line with the PRISMA-ScR within the ‘Conclusions’ subsection. We have also combined some of the paragraphs so that it's just five (in keeping with BMJ Open recommendations).<br><br>**Revision:**<br><br>***“Summary of evidence***<br><br>Our findings reveal multiple barriers and systemic shortcomings that contribute to experiences of discrimination and inadequate care for TGD people accessing healthcare in the UK. The strong association of positive experiences with individual clinicians demonstrates the impact of a compassionate, person-centred approach while pointing to inconsistencies in service delivery and care quality. Strategies for navigating healthcare reflect the resilience of TGD individuals and communities in the face of systems that frequently fail to provide appropriate support. Participant experiences spanned a range of healthcare services and settings, with care **related to gender transition** being most commonly explored. The predominance of qualitative studies reflects a focus on capturing nuanced lived experiences; however, the relatively small number of quantitative approaches indicates a lack of generalisable data.<br><br>***Limitations***<br><br>**Grey literature was included through reference list screening. However, as no formal grey literature search was conducted due to time and resource constraints, insights from non-academic and advocacy sources may be underrepresented. While** the selected databases covered multiple disciplines, searching additional databases and hand-searching key journals could have enhanced the comprehensiveness of the review. Evolving terminology around TGD topics also poses challenges in identifying all relevant literature**.** The review team included a queer genderqueer person, a gay cisgender man, and two heterosexual cisgender women.

We reflected on our individual and collective positionalities throughout, **with consideration of how this could influence the interpretation and synthesis of findings. We did not formally assess publication bias or study quality, which may limit the representativeness of our findings.**

Conclusions

To our knowledge, this is the first scoping review on the healthcare experiences of TGD people in the UK. Its national focus ensures direct relevance, reflecting experiences shaped by UK-specific healthcare policies, systems, and socio-cultural factors. Consistent with reviews by Holland et al. (10), Goulding et al. (15) and Chong et al. (16), our findings indicate that TGD people face widespread discrimination when accessing healthcare services. Inadequate knowledge among healthcare providers is consistently reported across the recent literature (10-16) and, as reflected in our review, often results in patients assuming the role of educator during healthcare interactions (10-12, 14, 16). **Taken together, these findings highlight persistent gaps between the care TGD patients report experiencing and the care required to meet their needs, underscoring the importance of improved and sustained professional education ~~to~~ addressing the specific health needs of TGD populations (10-15).**
Further research exploring barriers that **limit healthcare professionals' ability to effectively support TGD patients could contribute to the existing literature (83, 84**) **and** identify actionable strategies for service improvement. . Greater attention should also be given to under-explored contexts, such as pharmacy services, to develop a comprehensive understanding of the full spectrum of TGD healthcare needs. Additionally, existing qualitative insights could be supported by further quantitative research to provide statistical validation of commonly reported experiences.
The need for further research and inclusive **healthcare** practices is **particularly pressing** in light of the UK Supreme Court judgment in *For Women Scotland v The Scottish Ministers* (April 2025) (85). Although its full impact remains unclear at the time of writing**, the** judgment may have wide-ranging social and legal implications for the rights and recognition of TGD people in the UK (86). This **stresses** the immediate importance of generating robust, context-specific evidence to inform policy decisions that support gender-diverse populations, alongside ensuring that TGD people are meaningfully involved in **shaping** research and policies that directly affect them (87)."

VERSION 2 - REVIEW

**Reviewer**	**1**
**Name**	**Roth, Stephanie**
**Affiliation**	**ChristianaCare**
**Date**	**20-Jan-2026**

COI

I appreciate the work that the authors did to address the previous comments. The study limitations are now known and better addressed. This may still result in a lack of rigor in some areas of the search.

There seems to be more clarity needed in the following.

Since it is a scoping review, it would improve the review to add more than supplemental searching (citation chaining), with more focus on grey literature or stronger justification for not including.

Additionally, it is not clear what the librarian's role was in the search. The protocol states, "Search strategies were designed and refined with the assistance of a specialist librarian". What was the level of assistance? It seems like the librarian had a large role in the design making them eligible for authorship based on ICMJE, and advocacy work in this area for librarian author credit (https://www.mlanet.org/advocacy-resources/advocating-for-authorship/), as long as the librarian has rights to the content of the search and has consented to authorship.

Without more clarity in these areas, my recommendation of major revision, remains but I'd be happy to review it again when there are more explanations and transparency around the search and author credit. Thank you again for the opportunity to review your paper!

**Reviewer**	**2**
**Name**	**Ziegler, Erin**
**Affiliation**	**Ryerson University**
**Date**	**22-Jan-2026**
**COI**	

Thank you for addressing my comments.

VERSION 2 - AUTHOR RESPONSE

Reviewer 1:

Comment 1:

I appreciate the work that the authors did to address the previous comments. The study limitations are now known and better addressed.

Response:

Many thanks for acknowledging our revisions addressing your previous comments. We have also successfully resolved all comments from the other reviewer and the editors. We have now

addressed your additional minor comments and hope that the manuscript is now suitable for publication. Thank you again for your time and for your thoughtful comments.

Comment 2:
Since it is a scoping review, it would improve the review to add more than supplemental searching (citation chaining), with more focus on grey literature or stronger justification for not including.

Response:
We appreciate the reviewer's comment regarding grey literature. While we recognise its importance in scoping reviews, a formal grey literature search was not feasible due to time and resource constraints. To partially address this limitation, we screened the reference lists of all included studies to identify additional relevant sources beyond the indexed literature. We acknowledge that this approach may have missed unpublished or non-indexed evidence, including insights from non-academic and advocacy sources. The *Information sources & search* and *Limitations* sections have been revised to clarify this approach and to transparently acknowledge its potential impact on the comprehensiveness of the review.

Current:
(Information sources & search) Reference lists of included studies were subsequently searched to identify further relevant literature. No formal grey literature search was conducted.

(Limitations) Grey literature was included through reference list screening. However, as no formal grey literature search was conducted due to time and resource constraints, insights from non-academic and advocacy sources may be underrepresented. While the selected databases covered multiple disciplines, searching additional databases and hand-searching key journals could have enhanced the comprehensiveness of the review.

Revision:
(Information sources & search) **Due to time and resource constraints, a comprehensive grey literature search was not feasible. To partially address this,** reference lists of all included studies were screened **to identify additional relevant sources beyond the indexed literature.**

(Limitations) Grey literature was included through reference list screening. However, as no formal grey literature search was conducted, **unpublished or non-indexed evidence may have been missed. Specifically,** insights from non-academic and advocacy sources may be underrepresented, **which may limit the comprehensiveness of the review.** While the selected databases covered multiple disciplines, searching additional databases and hand-searching key journals **could have enhanced coverage.**

Comment 3:
Additionally, it is not clear what the librarian's role was in the search. The protocol states, "Search strategies were designed and refined with the assistance of a specialist librarian". What was the level of assistance? It seems like the librarian had a large role in the design making them eligible for authorship based on ICMJE, and advocacy work in this area for librarian author credit (https://www.mlanet.org/advocacy-resources/advocating-for-authorship/), as long as the librarian has rights to the content of the search and has consented to authorship.

Response:
We appreciate the reviewer's comment regarding the librarian's role. As described in the 'Information sources & search' section of the Methods, initial search strategies were developed

by the first and last author based on the research questions and previous reviews in the field. The librarian provided advisory assistance, directing us to the NICE UK filters to inform the geographical search terms, and subsequently reviewing the complete strategies to ensure that key concepts, terms, and search syntax were appropriately applied across databases. As such, the librarian's contribution does not meet ICMJE criteria for authorship. The librarian was aware that their contribution would not meet authorship criteria and that they would be acknowledged rather than listed as an author, which was understood and agreed in advance.
